# Allometric Scaling of Patrolling Rate and Nest Volume in *Constrictotermes cyphergaster* Termites: Hints on the Settlement of Inquilines

**DOI:** 10.1371/journal.pone.0147594

**Published:** 2016-01-25

**Authors:** Og DeSouza, Ana Paula Albano Araújo, Daniela Faria Florencio, Cassiano Sousa Rosa, Alessandra Marins, Diogo Andrade Costa, Vinicius Barros Rodrigues, Paulo Fellipe Cristaldo

**Affiliations:** 1 Laboratório de Termitologia, Departamento de Entomologia, Universidade Federal de Viçosa, Viçosa, MG, Brazil; 2 Laboratório de Interações Ecológicas, Departamento de Ecologia, Universidade Federal de Sergipe, São Cristóvão, SE, Brazil; 3 Departamento de Agrotecnologia e Ciências Sociais, Universidade Federal Rural do Semi-Árido, Mossoró, RN, Brazil; 4 Universidade Federal do Triângulo Mineiro, Iturama, MG, Brazil; 5 Departamento de Ciências Biológicas, Universidade do Estado de Mato Grosso, Tangará da Serra, MT, Brazil; Universidade de São paulo, BRAZIL

## Abstract

Structural and functional traits of organisms are known to be related to the size of individuals and to the size of their colonies when they belong to one. Among such traits, propensity to inquilinism in termites is known to relate positively to colony size. Larger termitaria hold larger diversity of facultative inquilines than smaller nests, whereas obligate inquilines seem unable to settle in nests smaller than a threshold volume. Respective underlying mechanisms, however, remain hypothetical. Here we test one of such hypotheses, namely, that nest defence correlates negatively to nest volume in *Constrictotermes cyphergaster* termites (Termitidae: Nasutitermitinae). As a surrogate to defence, we used ‘patrolling rate’, i.e., the number of termite individuals attending per unit time an experimentally damaged spot on the outer wall of their termitaria. We found that patrolling rate decayed allometrically with increasing nest size. Conspicuously higher patrolling rates occurred in smaller nests, while conspicuously lower rates occurred in larger nests presenting volumes in the vicinity of the threshold value for the establishment of inquilinism. This could be proven adaptive for the host and guest. At younger nest age, host colonies are smaller and presumably more vulnerable and unstable. Enhanced defence rates may, hence, prevent eventual risks to hosts from inquilinism at the same time that it prevents inquilines to settle in a still unstable nest. Conversely, when colonies grow and maturate enough to stand threats, they would invest in priorities other than active defence, opening an opportunity for inquilines to settle in nests which are more suitable or less risky. Under this two-fold process, cohabitation between host and inquiline could readily stabilize.

## Introduction

Body size affects structure and function of organisms and, hence, is of particular relevance for ecological and evolutionary processes. Traits ranging from morphology, physiology, and behaviour, to community ecology and biogeography are directly linked to some quantity (*e.g*. mass, volume) describing size in organisms in general [1–3], and in social insects [4–11] and termites [12–16] in particular. In colonial insects, such relationships are observed either with the size of individuals composing societies [4–6, 14, 16] or with the size of the colony itself [7–10, 12, 13, 15].

Adding to the growing list of size-dependent behaviours, heterospecific termite-termite cohabitation in a single nest, so called ‘inquilinism’, is also closely linked to the size of host termitaria. Larger as opposed to smaller termitaria are more prone to bear large diversity of inquilines [17]. Additionally, nest size can limit inquilinism even in those highly specialized cases (*i*.*e*. “obligatory inquilinism”, *sensu* [18]) where only a single inquiline colony cohabits with the termite host (e.g. *Constrictotermes cyphergaster* and *Inquilinitermes* spp.). In such cases, there is a minimum host nest volume below which inquiline colonies seem unable to settle in [19].

The reasons for positive relationships between nest size and inquilinism in termites are still under study and seem to be connected with nest defence strategies. Because builder individuals attack inquilines when they meet [20], it is highly likely that inquiline individuals will seek enemy-free spaces inside the mound. Being energetically demanding, defensive behaviours should correlate to metabolic rates which are known to decay allometrically (*i.e*. disproportionate) with size in whole termite nests [12]. It follows that defensive behaviours in termites should show decaying intensity with increments in nest size. Accordingly, nest volume has been hypothesized (rather than actually quantified) to correlate negatively to defence efficiency in termites based on the assumption that the higher load of termitophiles and inquilines typical of bigger termitaria would result from lesser defence [17]. Preliminary quantitative support for this hypothesis was recently provided by Cristaldo *et al*. [19]. Spotting the existence of critical volumes above which cohabitation was more likely to endure, these authors pushed forward the notion that rather than occurring purely at random, nest invasion obeyed constraints linked to colony growth and maturation.

Among these constraints, progressive relaxation in defence remains, to the best of our knowledge, an untested but sound hypothesis. Its soundness stems from both, the above empirical evidence and from theoretical trade-offs that are expected to happen between defence and reproduction as colonies maturate [21].

Here we test the hypothesis that nest defence correlates negatively to nest volume in nests of *Constrictotermes cyphergaster* (Silvestri, 1901) termites (Termitidae: Nasutitermitinae), aiming to shed light on still unanswered questions regarding processes and dynamics of inquilines’ settlement in termite nests. We inspected the correlation between nest volume and one facet of defence behaviour, namely ‘patrolling rate’, *i.e*., the number of termite individuals attending per unit time an experimentally damaged spot on the outer wall of these termitaria. We found that patrolling rate decayed allometrically with increasing nest size, and that conspicuously lower patrolling rates occurred at volumes in the vicinity of the threshold value for the establishment of inquilinism. We suggest that such an allometric decay would create an opportunity for the establishment of termite inquiline colonies in such nests, as earlier suggested [17, 19].

## Materials and Methods

### Ethics statement

All necessary permits were obtained for the described study, which complied with all relevant regulations of Brazil. This includes collecting and transportation permit (# 33094) from IBAMA (The Brazilian Institute for the Environment and Renewable Natural Resources), and permission from EMBRAPA (The Brazilian Enterprise for Agricultural Research) to conduct the study on their site. Tacit approval from the Brazilian Federal Government is implied by hiring ODS, APAA, DFF, CSR and DAC as Scientific Researchers, by awarding research grants to PFC, AM, VRB, and by awarding ODS with a Fellowship from CNPq (The Brazilian National Council for Research). No protected species was sampled.

### Terms definition and general rationale

We use the terms ‘termitarium’ and ‘nest’ to denote the physical structure built by termites [19, 22, 23]. ‘Colony’ denotes the assemblage of termite individuals living and cooperating within the nest. ‘Cohabitation’ and ‘inquilinism’ are used as synonyms and refer to the simultaneous occurrence of colonies of different termite species within a given termitarium, without implication of reciprocal positive or negative influences. The pair of termite species here studied is composed by the ‘host’, *Constrictotermes cyphergaster* and the ‘inquiline’, *Inquilinitermes microcerus* (Silvestri, 1901) (Termitidae: Termitinae).

We aimed to check likely connections between nest volume, nest defence efficiency, and inquilines’ settlement. Defence efficiency was estimated measuring how intensely would a colony react to sudden breaches on their nest wall. Colony’s reaction was estimated counting the number of soldiers and/or workers arriving at an experimentally disturbed point in the nest wall per unit time (hence, a ‘patrolling rate’). To shed light on the connection between nest defence and inquilinism, we inspected the changes (i) in patrolling rate and (ii) in the proportional contingent of nest primary defendants (*i.e*., soldiers) in the vicinity of the nest volume known as the threshold for inquiline’s settlement.

### Focal species

*Constrictotermes cyphergaster* is a common termite species in Brazil, Paraguay, Bolivia, and Northern Argentina [18], foraging at night in exposed columns and feeding mainly on wood, bark of live trees [24], and lichens [25]. In the study site, this species builds nests whose crumbly earthen walls (easily broken by the touch of a human finger) tend to get much harder with nest age. Nests are typically arboreal, but very young ones may be epigeous [26]. Active nests can harbour many other invertebrates, including one of two obligate inquilines, i.e., *Inquilinitermes microcerus* and *Inquilinitermes microcerus* and *Inquilinitermes fur* (Silvestri, 1901), and a large number of termitophiles, mainly Staphylinidae (Coleoptera). The settlement of cohabitants in these nests seems to be constrained by nest age or maturation: termitophiles and inquilines are more likely to be found in nests larger than 2.2 L and 13.6 L, respectively [19]. *Inquilinitermes microcerus* is believed to adopt furtive strategies to resolve its cohabitation in nests of *C. cyphergaster*. Among these, it feeds on a diet not used by its host [22] who, in addition, is not able to perceive this inquiline’s trail pheromones [23]. In spite of that, *I. microcerus* is perfectly able to perceive the pheromone trails laid by its host, *C. cyphergaster*. This mutual use of trail-following cues has been interpreted [23] as an additional means that the inquiline employs to evade its detection by the host within the nest.

### Study area

The study was carried out in the Brazilian *Cerrado*, an environment physiognomically but not floristically similar to a savannah, near the town of Sete Lagoas (19°27’ S, 44°14’ W, altitude 800–900 m above sea level), Minas Gerais State, South-eastern Brazil. According to Köppen’s classification, the study area is subjected to Aw climate (equatorial with dry winter) [27].

### Data collection

Procedures for data collection included (i) a main experiment to check for the correlation between nest volume and colony’s patrolling rate, and (ii) a population census to inspect the relative numbers of soldiers (“soldier density”) in a set of nests presenting volumes within the lower or upper neighbourhood to the threshold volume for inquiline’s settlement. Three density descriptors have been inspected: the number of soldiers relative to (i) the nest volume in litres, (ii) the total number of nestmates (workers + soldiers + alates) and (iii) the total number of workers.

The main experiment consisted of trials performed on 24 arboreal nests of differing sizes, while they were attached to their supporting tree. The choice of nests was made arbitrarily, in an attempt to sample nests in a continuous range of volume that broadly fitted the range of volumes available in the experimental area, as long as they were arboreal and did not show any sign of being connected to epigeous structures. Nest volumes were calculated based on the Cavalieri principle, by summing up the volumes of several superimposed cylindrical cross sections into which the nest was visually sliced, and the volumes of the hemispherical caps at both ends of the nest, as described and sketched by [19]. These trials were done in the beginning of the winter season (24 to 28 July 2008), during daylight from 7:30 a.m. to 2:00 p.m., when temperature varied from 17.0°C to 23.5°C never exceeding 5°C in a single day [28]. Termites are well known to thermoregulate their nests [29] and all of our bioassays have been conducted directly from nests in their normal state, still attached to the trees in the field (that is, termites have not been assayed in manipulative tests outside their normal settlement in their nest). Total precipitation in 2008 was 1469 mm, with 0 mm accumulated in July [30]. Only undamaged and active nests were inspected.

An experimental physical disturbance was made to a point located halfway between the nest’s base and top, using the stainless steel blade of a pocket knife to jab the nest on its exterior wall, forming a 3.5 × 1 cm (length×diameter) cylindrical hole. This blade was always cleaned with a new tissue before each trial. A wooden stick *c.a*. 20 × 0.5 cm (length × diameter) was immediately and partially inserted into this hole for 40 seconds, after which, clung worker and soldier termites were shaken into a plastic tray held right below the stick. For each trial, a new stick was used. Termites were collected from the tray with entomological forceps, placed in 80% ethanol, labelled, and taken to the laboratory for quantification and identification.

The waiting time of 40 s for the above trials was determined in a pilot test which inspected waiting times for the trials in the field, aiming to determine a trial timespan in which soldiers arrived to the disturbed point and climbed the stick but did not return to the nest. To do so, six nests (not used in the main experiment) were submitted to the disturbance procedure described above, each of them subjected to a trial lasting a distinct timespan, from 10 to 60 seconds after disturbance. Termite soldiers accumulated on the stick during each timespan were quantified as above.

Additionally, a population census was performed in eight other nests (not used in the main experiment nor in the pilot test) to inspect whether the density of soldiers in the nests would be affected by the threshold value for inquilinism (nest volume = 13.6 L, see [19]). To do so, we asked whether nests below this threshold volume would have more soldiers per unit volume, per nestmate, or per workers, than nests bigger than this. Nests presented volumes ranging from 5.6 to 24.3 L; four of these presenting volumes below the 13.6 L threshold and four being larger than it. This range is within the volume range of the 24 nests used to estimate patrolling rate, which varied from 0.4 to 41.4 L. Nests have been collected in the field and brought to the lab, where they were broken into pieces to extract their whole population for posterior estimation of its total numbers. This was done manually counting the number of individuals in a 10% aliquot of the volume comprised by all individuals of a given caste found in the nest. Total number of individuals in each caste in each nest was estimated extrapolating from the numbers counted in the aliquot. Such an aliquot is the double of that which was validated by Cunha *et al*. [31] to estimate populations in nest of *C. cyphergaster*.

These nests have been collected in the same Cerrado area as the ones used for the main experiment, in September 2012. Total precipitation in 2012 was 1067.4 mm, with 5.4 mm accumulated in September, when the temperature ranged from 9.7°C to 36.9°C, averaging 23.3°C in that month [30].

Termites were identified to species level, following Mathews [18]. Identifications were confirmed by comparison with the collection of the Isoptera Section of the Entomological Museum of the Federal University of Viçosa (MEUV), where voucher specimens were deposited.

### Data analysis

Statistical analyses proceeded in R [32] using Generalized Linear Modelling (GLM) under Poisson errors with log-link, corrected for overdispersion with quasi-likelihood function [33]. Subsequent residual analyses confirmed model suitability and the choice of error distribution. For the pilot test, analyses inspected the relationship between the number of soldiers arriving at the disturbed spot (y-var) and time elapsed after disturbance (x-var), hence establishing waiting times (as defined above) to carry out the main experiment. For the main experiment, the y-var ‘patrolling rate’ was defined as the number of individuals per second arriving at the disturbed spot. Modelling consisted in fitting to the data a decaying power-law function, using log-log transformations to ease parameter estimation. Analyses then inspected the effect of the nest volume (x-var) on this patrolling rate, considering either the total number of arriving individuals (soldiers + workers) or each of these castes in independent analyses.

Analyses included also a covariate to account for differences in disturbance intensity suffered by the nests as a consequence of the experimental method. This was needed because all tested termitaria had holes of the same dimension jabbed to their wall, which implies that larger termitaria would have suffered less disturbance per unit volume than smaller ones. This was estimated as the proportion of each nest volume actually destroyed by the jackknife blade, dividing the volume of the disturbance hole by the nest’s total volume. This was done in both, the pilot test and the main experiment.

Further analyses on the nest population census, inspected the effects of threshold nest volume (x-var), categorized as ‘below 13.6 L.’ and ‘above 13.6 L.’, on several variables describing soldier density in the nest (y-vars). These were the number of soldiers relative to (i) the nest volume, (ii) the sum of workers and soldiers and (iii) the number of workers found in the colony. To do so, Generalized Linear Modelling with normal errors was applied independently to each of the above y-vars. Suitability of the model error distribution were confirmed by subsequent residual analyses.

Raw data of all performed analyses are provided in Supporting Information (data in S1 Dataset).

## Results

### Validating the method

In the pilot test, the number of individuals arriving at the disturbed spots was not affected by the duration of the trial (*F*_[1, 4]_ = 0.023, *p* = 0.888) nor it was affected by the differences in the proportion of the nest destroyed by the jackknife blade (*F*_[1, 3]_ = 0.0003, *p* = 0.988). Such results supported the method as unbiased while allowing each trial to be run for the most practical waiting time in the field (40 seconds).

### Patrolling rate

Most of the individuals arriving at the disturbed spots were soldiers, with an average of 0.13±0.01 (mean±se) soldiers per second in each trial, compared to an average of only 0.01±0.001 (mean±se) workers arriving per second.

**Fig 1:**
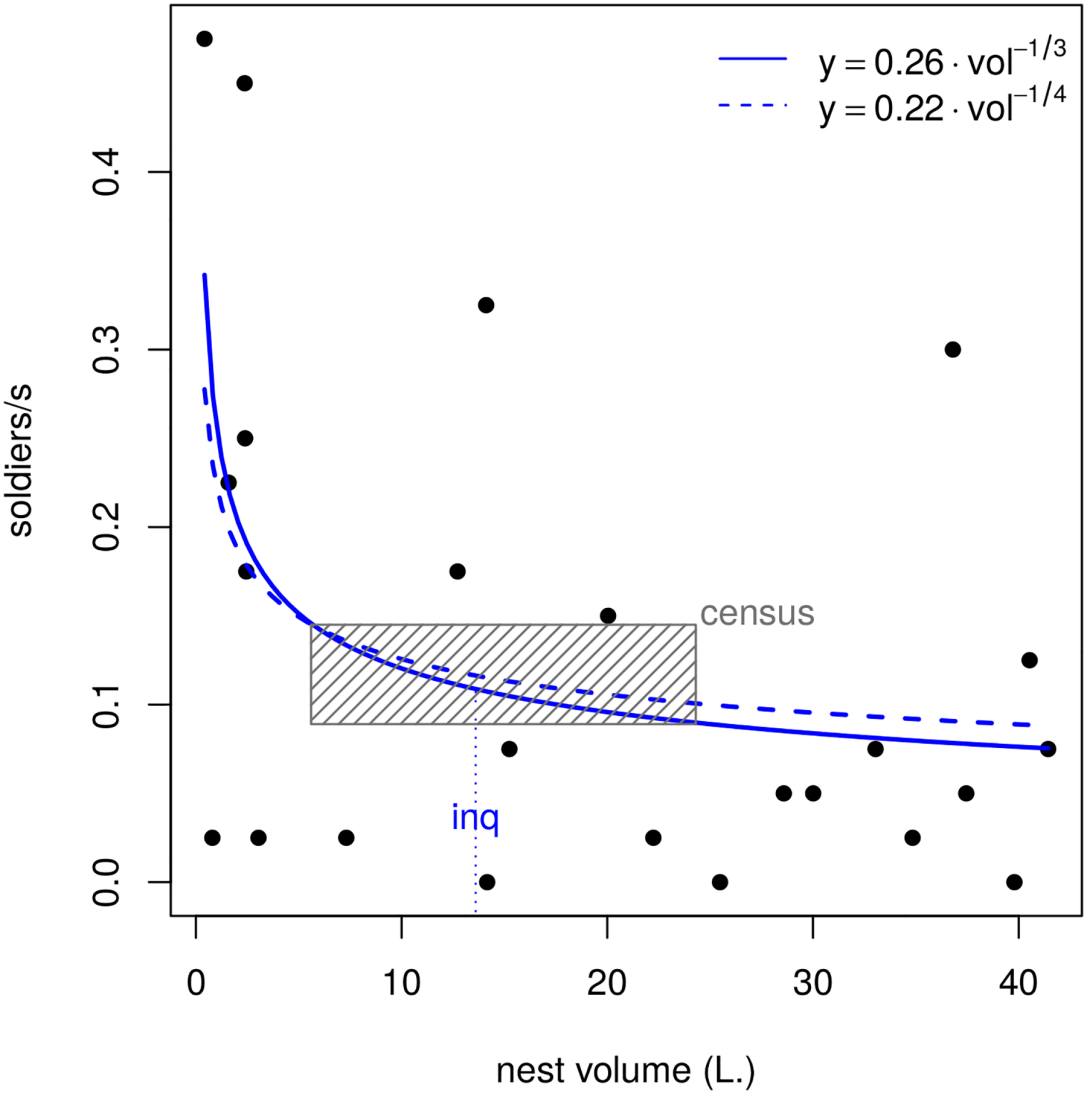
Decreasing numbers of soldiers, per second, attending a breach on the wall of nests of increasing volume in *Constrictotermes cyphergaster* termites. Each dot represents a single trial performed on a distinct nest. Curves denote distinct fits, each with a given exponent. Both curves differ significantly from the null hypothesis of no correlation (*p* < 0.05) and do not differ from each other (*p* = 0.781). The obligate inquiline *Inquilinitermes microcerus* is found in nests above 13.6 L (“inq”) [19]. Hatched rectangle marks the volume range of additional nests in which population census was performed, as depicted in Fig 2.

The number of soldiers attending a disturbed spot in the outer nest wall of *C. cyphergaster* termites decayed allometrically with nest volume (Fig 1, Table 1) being not affected by the differences in the proportion of the nest destroyed by the jackknife blade (*i.e*, ‘method’ in Table 1). Whatever the underlying mechanism, it was majorly due to soldiers, as correlations between individuals arriving at the disturbed spot and nest volume were significant for soldiers + workers (*p* = 0.015) and for soldiers only (*p* = 0.017) but not for the rate of workers only (*p* = 0.898).

**Table 1:** Effect of nest volume and experimental method on patrolling rates by *C. cyphergaster* termite individuals.

	Df	Deviance	Resid. Df	Resid. Dev	F	Pr(>F)
A) (Soldiers + Workers) per second						
Null model			23	3.02		
log(vol)	1	0.74	22	2.28	6.97	0.0153
method	1	0.01	21	2.27	0.06	0.8055
B) Soldiers per second						
Null model			23	3.25		
log(vol)	1	0.77	22	2.48	6.66	0.0175
method	1	0.01	21	2.47	0.10	0.7608
C) Workers per second						
Null model			23	0.67		
log(vol)	1	0.00	22	0.66	0.02	0.8983
method	1	0.03	21	0.63	0.72	0.4067

Patrolling rates were measured as the number of (A) soldiers and workers, (B) soldiers only, and (C) workers only, arriving per second at a cylindrical breach experimentally jabbed on the outer nest wall. The effects of the experimental method were estimated by the ratio between the volume of this breach and the volume of the nest. Generalized Linear Modelling under Quasipoisson errors.

Patrolling rate of *soldiers and workers taken together* obeyed a decaying function of the form

P=0.27v-13 (1)

where *P* is the patrolling rate (soldiers + workers, per second) and *v* is the nest volume in litres. Because the 95% confidence intervals of the exponent (−0.32) ranged from −0.23 to −0.40, this function did not differ (*F*_[1, 23]_ = 0.332, *p* = 0.57) from a more general power-law of the form:

P=0.24v-14 (2)

whose −14 exponent is known to describe many other biological rates [2, 34].

Similarly, the patrolling rate of *soldiers alone* obeyed a decaying function of the form:

P=0.26v-13 (3)

where *P* is the patrolling rate (soldiers per second) and *v* is the nest volume in litres. Its exponent (-0.33) presented 95% confidence intervals (−0.24 to −0.42) which also included the −14 exponent observed for all individuals taken together (workers + soldiers, Eq 2 above). This equation for ‘soldiers only’ did not differ (*F*_[1, 23]_ = 0.424, *p* = 0.521) from a more general power function of the form (Fig 1):

P=0.22v-14 (4)

### Nest population census

Total population censused in nests was highly variable, ranging from 3,917 to 36,903 individuals and averaging 14,281±3,918.8 (mean±se). As expected, all bigger nests (volumes > 19 L) held inquilines while they were absent from two smaller nests (volumes = 10.3 and 12.1 L). Such a pattern (inquilines being linked to bigger and not to smaller nests) was confirmed statistically (GLM with binomial errors: *χ*^2^ = 11.09, 1 df, *p* < 0.001). This apparently supports the idea for an inquilinism threshold located at some point in the range from 12 to 19 L, but since we censused only eight nests, it would not be reliable to apply the necessary logistic regression to confirm this threshold. Because these nests are inhabited by the same pair of host-inquiline species and have been collected at the same site as those used by Cristaldo *et. al*. [19], we conformed to their previous published threshold of 13.6 L as a reference for propensity to inquilinism.

**Fig 2:**
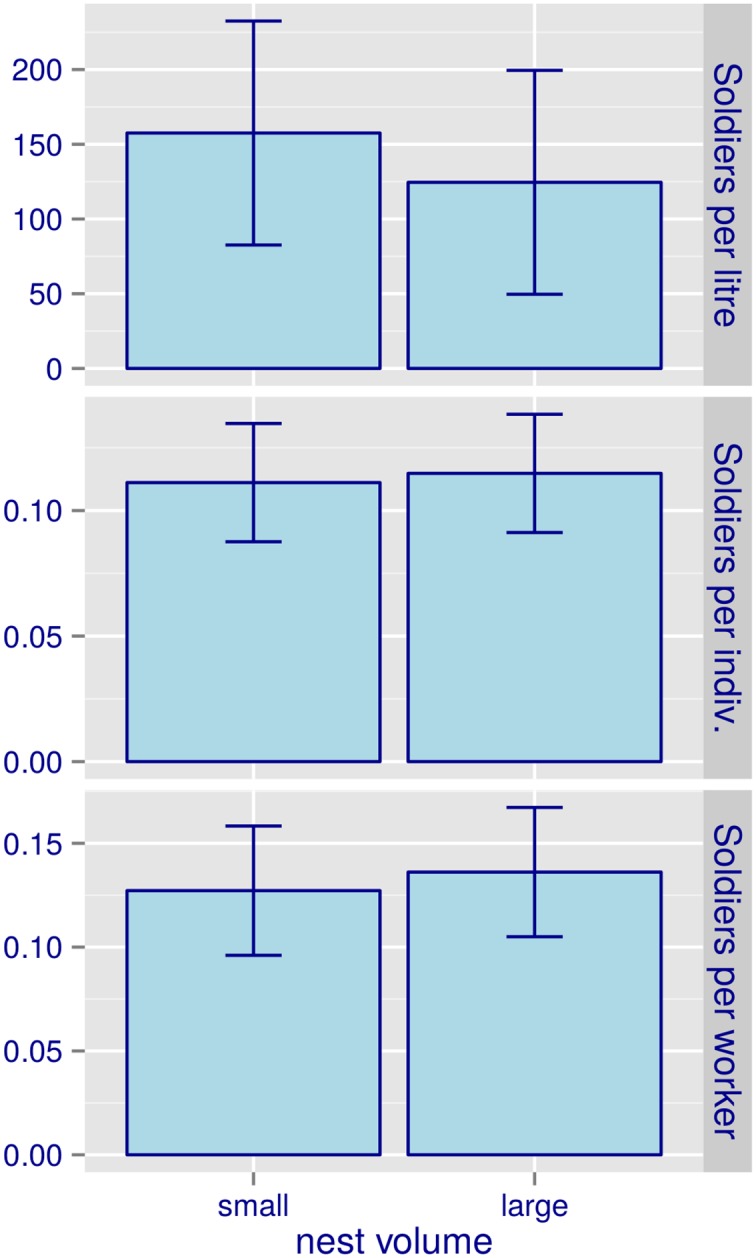
Soldier density relative to (top to bottom) (i) total nest volume, (ii) total number of soldiers plus workers, and (iii) total number of workers, in *C. cyphergaster* nests. Bars refer to classes of nest size relative to the threshold value for the establishment of obligate inquilines [19]. Small nests: volume < 13.6 L. Large nests: volume > 13.6 L. This range of nest volumes is depicted as a hatched rectangle in Fig 1.

In such nests, the density of soldiers seemed not related to the volume threshold for inquilinism: the number of soldiers per litre, the number of soldiers relative to the sum of soldiers plus workers, and the number of soldiers relative to the sum of workers, did not differ between nests whose volume was smaller than the inquilinism threshold (13.6 L) and nests bigger than that (Table 2 and Fig 2). This seems to indicate that higher defence rates in smaller nests (Fig 1) would have arisen from prompt response by soldiers, rather than from a disproportionately higher contingent of soldiers in such nests.

**Table 2:** Effects of nest size on several descriptors of the density of soldiers in *C. cyphergaster* colonies.

	Df	Deviance	Resid. Df	Resid. Dev	F	Pr(>F)
(A) Soldiers per litre						
null model			7	136887.42		
nest size	1	2175.56	6	134711.86	0.10	0.7661
(B) Soldiers per nestmate						
null model			7	0.01		
nest size	1	0.00	6	0.01	0.01	0.9158
(C) Soldiers per worker						
null model			7	0.02		
nest size	1	0.00	6	0.02	0.04	0.8457

Nests have been categorized into two sizes, small and large, relative to the threshold value of 13.6 L above which they are more likely to house inquilines, at least at this geographic region [19]. Densities refer, in a given nest, to the total number of soldiers relative to (A) nest volume in litres, (B) total number of nestmates (soldiers, workers, and alates taken together), and (C) total numbers of workers. Generalized Linear Modelling under Normal errors.

## Discussion

Allometric relationships involving body size are pervasive: they affect almost all biological traits across taxonomic and functional groups, from molecular to whole-organism level, from eukaryotes to plants and mammals, and from herbivores to omnivores and carnivores [2, 35–37]. Additionally, theoretical assumptions regarding size dependence of biological traits of unitary organisms can be equally applied to whole colonies of social insects [38].

Here we detected an inverse allometric scaling between nest volume and patrolling rate by termite soldiers. Lower patrolling rates occurred at nest volumes similar to those in which inquilines are more likely to settle in *C. cyphergaster* termitaria (Fig 1, Table 1). This result suggests a somewhat composite effect of allometry. Decay in patrolling rates seems to denounce waning vigilance as termite nests grow, which in turn would favour the hypothesis of lesser defence favouring settlement of inquilines in bigger nests, as suggested by Domingos [17].

Two distinct (hypothetical) mechanisms could have produced such a decay: as compared to bigger nests, smaller nests could hold either (i) proportionately more soldiers or (ii) more active soldiers. Available evidence does not point to the first of these mechanisms, since unchanged soldier proportions (individuals/volume) as nest grows seem, in fact, to be the norm for *C. cyphergaster*. It was already observed by Cunha *et al*. [31] who detected unchanged soldier proportions (individuals/volume) for this same species in nests whose volumes (0.6 to 69 L) overlapped the range of volumes here studied. Accordingly, in the additional set of nests here studied, soldier proportions (relative to nest volume, to soldiers + workers, or to workers) did not differ between small and large nests (Fig 2, Table 2). It seems thus that decay in patrolling rate here observed could not be imputed to a corresponding decay in the proportional amount of soldiers in larger nests. Because neither of the above results refer directly to the nests on which we have measured patrolling rates, this conclusion remains open to investigation.

The second hypothetical mechanism, on the other hand, seems better supported. Observed differences in patrolling rates could derive from changes in soldiers activity according to nest size. Changes in body size, indeed, are known to lead to distinct behavioural traits in vertebrates [39] and invertebrates [4, 40]. Accordingly, higher promptness in smaller nests, are perfectly possible, e.g., as a consequence of the fact that in smaller nests the core is relatively closer to outer edges. This would allow defenders to quickly arrive at any point of the nest surface no matter where they were at the stimulus’ onset. This could be reinforced by alarm calls being readily perceived in smaller as opposed to bigger nests so that more individuals could be recruited *per* unit time. Additionally, frenzy building and growth typical of young/smaller nests should imply in speedy movements of nestmates inside nests and, hence, reflect in prompt patrolling. Adaptiveness of such a mechanism can be inferred from the fact that younger (hence, smaller) colonies, being in the beginning of their establishment, are more vulnerable and need more prompt defence.

Lower promptness of termite soldiers in bigger nests are equally possible and may occur either alternatively or complementary to higher promptness in smaller nests. Diminished patrolling by soldiers in larger nests could result, for instance, from a shift in strategy from active defence by soldiers to passive defence owed to physical nest structure. Bigger and presumably older nests in this species tend to present harder walls (ODS and PFC, pers.obs.; see Material and Methods), hence standing damage from accidents or predators. Such a shift in defence strategy could be proven adaptive because, differently from soldier contingent, harder walls are stable across seasons and therefore less expensive in the long run. This is amplified by the fact that any given attack is relatively smaller in bigger nests and, hence, proportionately less harmful in larger colonies as opposed to smaller ones. Consequential lowered frequency and intensity of alarm should lead to decay in defensive reaction [41]. Soldiers, being an expensive caste, would be kept in larger nests as if a military reserve for severe emergencies, or be diverted to other tasks. This is in line with Evans [42] who reported the existence of a reserve worker force which is activated in emergencies in *Nasutitermes exitiosus*, a species belonging to the subfamily Nasutitermitinae, like *C. cyphergaster*, the host termite here studied. Larger colonies, therefore, would be selected to take ‘calculated risks’, saving energy by combining active and passive defence.

This hypothesis of optimal use of energy seems to be further supported by a mathematical feature of the power-law decay in defence rate. Its scaling exponent did not differ from −14 (*p* = 0.781), which is thought to derive from optimal use of energy to distribute resources over the entire volume of a space filling fractal-like branching pattern ([37, 43, 44]; but see [45]) typical of galleries within termite nests [46]. In other words, as already proposed in the context of *Nasutitermes ephratae*, ‘it seems safe to assume that termite colonies optimize their use of energy as colonies get larger’ [12].

As a side-effect, however, decrements in patrolling rate seem to provide opportunities for inquilines to colonize the nest, minimizing risks of fierce contention with host defendants. That is, inquilines could sneakily profit from allometric scaling in *C. cyphergaster*, if they learn the precise moments when a plastic phenotype of the host will turn it into a susceptible or suitable phenotype. Such a moment, previously predicted to occur as nests attain 13.6 L [19], corresponds to the volume around which patrolling rates were detected here as severely depressed (Fig 1).

Inquilines here studied, being obligatory and specific to this host, are expected to adopt strategies to minimize eventual conflicts with their host. By avoiding overlaps in diet with their host [22] and eavesdropping their host chemical cues [23], *I. microcerus* is believed to avoid confrontation hence lowering eventual costs of its acceptance by *C. cyphergaster*. As a consequence, this particular case of inquilinism would not pose strong selective pressure against decrements in patrolling rate in this host. Since other termite inquilines are hardly ever found cohabiting with this host, it is safe to assume that inquilinism in *C. cyphergaster* would not counteract, per se, whatever benefits are associated to decrements in patrolling rate in this case.

In short, inverse allometric scaling of patrolling rates with nest size seem to be advantageous for *C. cyphergaster* and also for its inquiline, *I. microcerus*. At younger nest age, when host colonies are smaller and presumably more vulnerable and unstable, enhanced rates may prevent eventual risks to hosts from inquilinism at the same time that it prevents inquilines to settle in a still unstable nest. In older (larger) nests, when host colonies are well established, general principles of energy optimization would lead to decayed patrolling rates. This would, then, create opportunity for inquilines, which would be able to colonize a host colony which is more suitable or less risky.

More than simply reporting a new example of allometric links between body size and behaviour, here we show a somewhat composite effect: nest size affects patrolling rate which in turn potentially affects inquilinism in termites. We anticipate these findings to reach a broader perspective, because inquilinism provides insights into a suite of interactions including self/non-self recognition and tolerance-resistance adjustment, which are relevant to themes as diverse as sociality, immunity, and host-parasite interactions.

## Supporting Information

S1 Dataset Raw data used in all performed analyses. (ZIP)
